# PEDIATRIC APPENDICITIS: AGE DOES MAKE A DIFFERENCE

**DOI:** 10.1590/1984-0462/;2019;37;3;00019

**Published:** 2019-06-19

**Authors:** Belén Aneiros, Indalecio Cano, Araceli García, Pedro Yuste, Eduardo Ferrero, Andrés Gómez

**Affiliations:** a Hospital 12 de Octubre, Madrid, Spain.

**Keywords:** Appendicitis, Child, Laparoscopy, Apendicite, Criança, Laparoscopia

## Abstract

**Objective:**

To investigate the influence of patient age on the diagnosis and management of appendicitis, as well as to evaluate the rate of complications according to the age group.

**Methods:**

We undertook a retrospective analysis of 1,736 children who underwent laparoscopic appendectomy in our center between January 2000 and December 2013. Patients were divided in groups taken into account their age: group A were infants, group B were preschoolers, group C were those ones older than five years old, and group D were those ones younger than five years old. A p value of 0.05 was considered statistically significant.

**Results:**

We found higher incidence of misdiagnosis and atypical symptoms in the youngest patients. The rate of perforation was similar between group A and B (p=0.17). However, it was higher in group D than in group C (p<0.0001). The incidence of postoperative complications was higher in the youngest patients too (p=0.0002).

**Conclusions:**

The age does make a difference in acute appendicitis. Because of its unusual presentation in children younger than five years old, it is often misdiagnosed, which leads to an increased morbidity. Although clinical presentation varies between infants and preschoolers, no statistically significant differences were observed in the rate of perforated appendix or postoperative complications.

## RESUMO

**Objetivo:**

Investigar a influência da idade do paciente no diagnóstico e tratamento de apendicite, bem como avaliar a frequência de complicações dependendo da faixa etária.

**Métodos:**

Análise retrospectiva dos 1.736 pacientes pediátricos que foram submetidos à apendicectomia laparoscópica em nosso hospital de janeiro de 2000 a dezembro de 2013. Os pacientes foram divididos em grupos de acordo com sua idade: grupo A eram crianças, grupo B eram pré-escolares, grupo C eram maiores de cinco anos de idade e grupo D eram menores de cinco anos de idade. Considerou-se estatisticamente significante p-valor <0,05.

**Resultados:**

Encontramos maior incidência de diagnóstico incorreto e sintomas atípicos em pacientes mais novos. A taxa de perfuração foi semelhante entre os grupos A e B (p=0.17); foi maior, porém, no grupo D que no grupo C (p<0.0001). A incidência de complicações no pós-operatório também foi maior em pacientes mais novos (p=0.0002).

**Conclusões:**

A idade faz diferença em casos de apendicite aguda. Por causa da sua apresentação rara em crianças menores de cinco anos, é frequentemente diagnosticada incorretamente, o que aumenta a morbidade. Apesar de sua apresentação clínica variar entre lactentes e pré-escolares, não foram observadas diferenças estatisticamente significativas na proporção de apêndices perfurados nem na de complicações pós-operatórias.

## INTRODUCTION

Acute appendicitis is one of the most common surgical causes of acute abdominal pain in pediatric patients. The peak incidence is considered between the first and the second decade of life.^1^ Although it is rarely considered in children younger than five years of age, it can occur even in newborns. Prenatal cases have also been described.^2^

The classic presentation of appendicitis is the onset of periumbilical pain that migrates to the right lower quadrant followed by low-grade fever and nausea or vomiting. In children, particularly in the younger ones, these symptoms are infrequent and occur in less than 50% of the patients.^2^ ^,^ ^3^ Because of its atypical clinical features, misdiagnosis in preschool-age children ranges from 19 to 57%, which entails a high rate of complications.^2^

Although the improvements on the quality of care and the development of enhanced diagnostic tools, appendicular rupture continues to be a common occurrence in the young children.^4^ An early and accurate diagnosis in infants is difficult because of the limited communication and the variability in clinical course.^1^ ^,^ ^2^ ^,^ ^3^ ^,^ ^4^

The objective of this study was to investigate the influence of patient age on the diagnosis and management of appendicitis, as well as to evaluate the rate of complications according to the age group.

## METHOD

In the 14-year period from 2000 to 2013, 1,736 pediatric patients aged 15 years old or less underwent laparoscopic appendectomy for acute appendicitis at our tertiary center. Incidental, interval and negative appendectomies were excluded from this study. A retrospective analysis was carried out, and patients were subsequently divided into four groups according to their age: group A were infants-toddlers (0-2 years), group B were preschoolers (3-5 years), group C were those ones older than five years old; and group D were those ones younger than five years old (group A+B). For the purposes of this review, the groups were compared as follows: group A with group B and group C with group D.

Demographics, preoperative, intraoperative and postoperative data were collected from patients’ medical records. Preoperative parameters included duration of symptoms, physical signs and symptoms at presentation, previous misdiagnosis and ultrasound findings. Intraoperative variables included surgical time, operative findings, laparoscopic techniques, intraoperative complications and conversion to open surgery. Postoperative data were length of stay (LOS), antibiotic therapy, analgesic therapy, onset of oral intake and postoperative complications.

Previous misdiagnosis was considered to have occurred if patients had presented to a physician and an alternate diagnosis was indicated. Fever was defined as any temperature higher than 38.0ºC. An appendix diameter larger than 6 mm was considered pathological. Surgical trainees under direct supervision of pediatric surgeons operated the majority of the cases. Multiport laparoscopic-assisted appendectomy was the standard treatment in our center. However, surgical technique was chosen according to the surgeon’ preference. Perforated appendicitis was defined as an identifiable macroscopic hole in the appendix during the surgery. In cases of complicated appendicitis (gangrenous and perforated appendicitis), either ertapenem or intravenous triple antibiotics (ampicillin, gentamycin and metronidazole) were administered for at least 5-7 days postoperatively. In cases of non-complicated appendicitis, patients received at least one day of intravenous amoxicillin plus clavulanic acid therapy.

Because the data were not normally distributed, non-parametric test were used to compare the groups. Continuous variables were analyzed using the Wilcoxon test, while categorical variables were compared with the chi-square test. Missing data were left out of the analyses. All statistical studies were performed using the software package Statistical Analysis System (SAS) 9. Results showing p less than 0.05 were considered significant. The ethics committee of our hospital approved the study.

## RESULTS

There were 50 patients (2.9%) in the group A with a median age of 1.72 years (range: 0-2 years), 233 (13.4%) in the group B with a median age of 3.98 years (range: 3-5 years), 1,453 (83.7%) in the group C with a median age of 9.92 years (range: 6-15 years), and 283 in the group D with a median age of 3.58 years (range: 0-5 years). There was no difference in gender distribution between the groups. There were 32 males and 18 females in the group A and 131 males and 102 females in the group B (p=0.312). On the other hand, there were 925 males and 528 females in the group C and 163 males and 120 females in the group D (p=0.053).

In the first part of this study, we compared the parameters of the group A with those ones of the group B. Preoperative symptoms and physical signs of both groups are presented in Table 1. Vomiting and fever were the most common symptoms in both groups. There was no statistically significant difference in vomiting, urinary and respiratory symptoms. The most common physical sign was abdominal pain in both groups. However, unlike group B, group A developed more frequently diffuse abdominal pain (p=0.001). Patients in the group A presented the average of 2.68 days following the onset of symptoms compared with 1.68 days for those ones in the group B (p=0.01). Children sought prior medical care in eight cases (16%) in the group A and in 37 cases (15.8%) in the group B (p=0.97). Gastroenteritis was the most common misdiagnosis in both groups (five cases in group A and 12 cases in group B, p=0.18). Ultrasound findings showed a pathological appendix in 39 cases (84.8%) of the group A and in 185 cases (85.7%) of the group B (p=0.12).

**Table 1:** Comparison of the symptoms and signs between the groups A and B.

	Group A (n=50)	Group B (n=233)	p-value
Vomiting	32 (66.7%)	172 (74.5%)	0.267
Diarrhea	17 (35.4%)	35 (15.2%)	0.001*
Fever	39 (81.3%)	136 (58.9%)	0.009*
Urinary symptoms	2 (4.2%)	15 (6.5%)	0.535
Respiratory symptoms	7 (14.9%)	29 (12.7%)	0.719
Abdominal pain			
Focal	18 (39.1%)	148 (66.7%)	0.001*
Diffuse	21 (45.7%)	60 (27%)	
Ill-defined	7 (15.2%)	14 (6.3%)	
Tenderness			
Focal	11 (23%)	85 (38.1%)	<0.001*
Diffuse	17 (35.4%)	34 (15.3%)	
Ill-defined	20 (41.6%)	104 (46.6%)	

*Statistically significant.

Perforated appendicitis was found in 11 patients (22.5%) in group A and in 47 patients (20.2%) in group B (p=0.17). The preferred technique was a multiport laparoscopic-assisted appendectomy in both groups (p=0.77). There was no statistically significant difference in the surgical time between the groups (group A: 55.9 min; and group B: 53.5 min, p=0.74). Intraoperative complications occurred in three patients (6%) in the group A and in 28 patients (12%) in the group B (p=0.21). The rate of conversion was 2% in the group A and 0.43% in the group B (p=0.22).

Postoperative variables were shown in Table 2. The average of antibiotic and analgesic therapy, time to resumption of oral intake and LOS were statistically higher in the group A. No significant differences were observed in the rate of postoperative intra-abdominal abscess, wound infection and bowel obstruction between both groups. No deaths occurred during the study period.

**Table 2:** Comparison of the postoperative variables between the groups A and B.

	Group A (n=50)	Group B (n=233)	p-value
Antibiotic therapy (days)	5.94	4.53	0.003*
Onset of oral intake (days)	2.59	1.86	0.002*
Analgesic therapy (days)	5.42	3.36	0.004*
Length of stay (days)	9.38	6.41	0.006*
Intra-abdominal abscess	8 (16%)	28 (12%)	0.443
Wound infection	0	7 (3%)	0.214
Bowel obstruction	1 (2%)	6 (2.6%)	0.812

*Statistically significant.

In the second part of our study, we made a comparison between groups C and D. The symptoms and the physical findings of both groups are presented in Table 3. Vomiting, diarrhea, fever and respiratory symptoms were significantly higher in the group D. Diffuse abdominal pain was also observed more frequently in the group D than in the group C (30.2 and 10.4%, respectively), as well as diffuse tenderness (18.8 and 5.9%, respectively). Patients in the group C had a mean duration of symptoms of 1.14 days, while those ones in the group D had the mean duration of symptoms of 1.85 days (p<0.001). One hundred and thirty children (8.9%) in the group C and 45 children (15.9%) in the group D had previously been examined without a correct diagnosis (p=0.0003). Gastroenteritis was the most common misdiagnosis in both groups (37 patients in the group C and 17 patients in the group D, p=0.001). Ultrasound findings suggestive of appendicitis were found in 1,237 cases (89.1%) of the group C and in 224 cases (85.5%) of the group D (p=0.08).

**Table 3:** Comparison of the symptoms and signs in the groups C and D.

	Group C (n=1,453)	Group D (n=283)	p-value
Vomiting	954 (66.2%)	204 (73.1%)	0.024*
Diarrhea	181 (12.6%)	52 (18.6%)	0.007*
Fever	484 (33.5%)	175 (62.7%)	<0.001*
Urinary symptoms	110 (7.6%)	17 (6.1%)	0.377
Respiratory symptoms	42 (2.9%)	36 (13%)	<0.001*
Abdominal pain			
Focal	1,221 (84%)	166 (58.6%)	<0.001*
Diffuse	147 (10.1%)	81 (28.6%)	
Ill-defined	29 (2%)	18 (6.4%)	
Doubtful	56 (3.9%)	18 (6.4%)	
Tenderness			
Focal	514 (35.4%)	96 (34%)	<0.001*
Diffuse	83 (5.7%)	51 (18%)	
Ill-defined	30 (2.1%)	19 (6.7%)	
Doubtful	826 (56.8%)	117 (41.3%)	

*Statistically significant.

The rate of appendiceal perforation was inversely proportional to patient age, occurring in 9.7% of cases in the group C and in 20.6% of cases in the group D (p<0.001). The standard technique was employed more frequently in the group D (91 *versus* 88.7%, p=0.02) and the surgical time was higher in the group C (58 *versus* 54 min, p=0.02). The rate of intraoperative complications and conversion was also higher in the group C, though not significantly so (14.5 *versus* 11% and 1 *versus* 0.7%, p=0.11 and p=0.60; respectively).

Postoperative outcomes and complications were summarized in Table 4. The average of antibiotic and analgesic therapy, onset of oral intake and LOS were statistically higher in the group D. Postoperative intra-abdominal abscess occurred in 6% of patients in the group C and in 12.7% of those ones in the group D (p<0.001). However, the rates of wound infection and bowel obstruction were not affected by age. There was no death in these groups.

**Table 4:** Comparison of the postoperative outcomes and complications between the groups C and D.

	Group C (n=1,453)	Group D (n=283)	p-value
Antibiotic therapy (days)	3.17	4.77	<0.001*
Onset of oral intake (days)	1.39	1.99	<0.001*
Analgesic therapy (days)	2.39	3.73	<0.001*
Length of stay (days)	4.59	6.94	<0.001*
Intra-abdominal abscess	87 (6%)	36 (12.7%)	<0.001*
Wound infection	25 (1.7%)	7 (2.5%)	0.389
Bowel obstruction	19 (1.3%)	7 (2.8)	0.139

*Statistically significant.

## DISCUSSION

Acute appendicitis is the primary cause of abdominal surgical interventions in emergency departments in pediatric and adult ages.^1^ Although it is very common in children, it is rare in infants and even more in neonates.^2^ ^,^ ^3^ ^,^ ^4^ Some authors have attributed the low incidence in this population to the lack of prominent lymphoid tissue in infancy.^1^ ^,^ ^2^ The rates of appendicitis in our series was 2.9% in infants/toddlers and 13.4% in preschoolers, which are higher than those reported in other studies.^5^ ^,^ ^6^ This is probably because our institution is a reference center for pediatric surgery.

It has been supported that there is a faster inflammation of the appendix in the youngest patients.^7^ ^,^ ^8^ ^,^ ^9^ Furthermore, variations in appendiceal development according to the patient age could explain differences in the progression of the disease. The thin-walled appendix and the inappropriate barrier function of the omentum in the youngest patients may lead to a rapid spread of the infection.^8^ ^,^ ^9^ In addition, infants has a small appendiceal lumen. Therefore, the critical pressure to achieve perforation is obtained earlier than in adults.^9^

A fast and accurate diagnosis in pediatric patients is not straightforward. Children younger than two years old are not able to express well what their complaints are; and consequently, their caregivers must complete the medical history. Infants cannot localize the pain at all and they are frequently uncooperative patients, which complicates physical examination.^4^ ^,^ ^9^ Moreover, it is not uncommon the presence of symptoms that can mimic more prevalent diseases at this age, such as gastroenteritis, urinary tract infections, gynecological pathology, upper respiratory tract infections or inguinal canal disorders.^2^ ^,^ ^5^ This entails the risk of misdiagnosis and delayed diagnosis. Many researches have highlighted the elevated rate of inadequate diagnosis in this population.^1^ ^,^ ^2^ ^,^ ^3^ ^,^ ^5^ ^,^ ^10^ In our series, we have observed a lower rate of misdiagnosis (range: 9.1-16.9%) compared with other previous series. However, we feel that it is still too high, especially in the youngest patients. As already demonstrated, gastroenteritis was the most common incorrect diagnosis.^10^ It has been reported that delays in diagnosis are associated with patient age.^10^ ^,^ ^11^ We agree that the younger the patient, the higher the mean duration of symptoms.

By comparing the symptoms of patients with appendicitis among different age groups, we observed that diarrhea and fever were statistically more frequent in infants/toddlers than in preschoolers. Similarly, vomiting and respiratory symptoms, as well as diarrhea and fever, were more common in patients younger than five years old than those ones older than that. These results are in agreement with other studies in which the authors emphasize the presence of unusual symptoms such as diarrhea or respiratory symptoms in youngest patients.^12^ ^,^ ^13^ ^,^ ^14^ Small children also have a higher rate of diffuse abdominal pain and tenderness.^2^ ^,^ ^15^

Developments in health care have improved the outcomes of appendicitis. A significant drop in mortality has been observed in the last decades, especially in newborns.^16^ ^,^ ^17^ However, the rate of perforated appendicitis has remained almost unchanged. Appendiceal perforation in children younger than five years old may occur in more than half of the patients.^18^ ^,^ ^19^ In our study, the rate of perforation in children younger than five years old was 20.6%, which is in line with the mean duration of the symptoms and the rate of misdiagnosis in these patients. Perforated appendicitis could explain a greater frequency of diarrhea and respiratory symptoms in this population due to peritonitis can produce an irritation of the colon and a diaphragmatic elevation.^2^

Laparoscopic appendectomy has potential advantages compared to the open surgery, which have even been demonstrated in newborns.^20^ ^,^ ^21^ However, postoperative outcomes depend not only on a meticulous surgical technique. The development of complications are strongly associated with delays in diagnosis and, therefore, with patient age.^22^ Intra-abdominal abscesses are the major complication and they are associated with increased morbidity.^23^ In our study, the incidence of intra-abdominal abscess was statistically higher in patients younger than five years old. Nevertheless, this difference did not exist between infants/toddlers and preschoolers. The duration of antibiotic and analgesic therapy, the onset of oral intake and the LOS were also higher in patients younger than five years old, but in this case these parameters were higher in infants/toddlers compared to preschoolers. With these results, it seems that postoperative evolution depends as much on the age, duration of symptoms and misdiagnosis as on the rate of appendiceal perforation and widespread peritonitis.

Laboratory tests such as absolute neutrophil count (ANC) and white blood cell (WBC) count have long been used in the diagnosis of appendicitis. However, their value in the clinical decision-making process still remains unclear. Several studies to assess the sensitivity and specificity of these biomarkers have been performed in the last years.^24^ ^,^ ^25^ A multicenter prospective study has demonstrated that the diagnostic performance of WBC and ANC improves with increasing age.^25^ Moreover, ultrasound is becoming increasingly popular in the diagnosis of appendicitis, and its overall diagnostic accuracy is satisfactory.^26^ ^,^ ^27^ We observed that ultrasound findings suggestive of appendicitis do not vary by age. Misdiagnosis rates may be improved if we perform an early ultrasound in case of suspicion of appendicitis in young children, regardless the value of biomarkers.

The interpretation of the results of this study is limited by its no randomized and retrospective nature. One of the study limitations is the fact that ultrasound findings are operator dependent, as well as physical exploration. However, we found some differences due to the large sample size that provided us detailed data.

In conclusion, the age does make a difference in acute appendicitis. Because of its unusual presentation in children younger than five years old, it is often misdiagnosed, which leads to an increased morbidity. Although clinical presentation varies between infants and preschoolers, no statistically significant differences were observed in the rate of perforated appendix or postoperative complications. Future efforts must be focused on minimizing delays in diagnosis to avoid appendiceal perforation and its potential complications. An early ultrasound may be the key to prevent misdiagnosis and to achieve the accurate diagnosis.
